# Supplementary material for: Mitochondrial Genome Variations and Possible Adaptive Implications in Some Tephritid Flies (Diptera, Tephritidae)
Source: Int J Mol Sci. 2025 Jun 10;26(12):5560. doi: 10.3390/ijms26125560 (PMC12193529; doi:10.3390/ijms26125560)
Supplement: Supplementary file 1 [file ijms-26-05560-s001.zip › Supplementary Data Legends.pdf]

Supplementary Data

Supplementary Table S1. Nucleotide composition Tephritidae. Composition including extracted sequences (*), Ns indicate percentage of ambiguities throughout the sequences.

Supplementary Table S2. Dataset information and features for extracted mitogenomes: Taxonomic identification, NCBI SRA ID, Assembly reference, total and assembled reads, average coverage and NCBI accession.

Supplementary Table S3. Mitogenome architecture of analyzed species of Tephritidae. Taxonomic identification, NCBI ID and gene order ranging from 1 to 37 according to synteny proposed by Cameron, (2014).

Supplementary Table S4. Nucleotide composition and asymmetry values of AT and GC. Values for full genome (Sheet 1), concatenated protein coding genes (Sheet 2), individual coding genes (Sheet 3) and concatenated transfer RNAs (Sheet 4).

Supplementary Table S5. Relative Synonymous Codon Usage (RSCU) values for each codon ordered by aminoacid in analyzed Tephritidae species.

Supplementary Table S6. Codon Adaptation Index (CAI) values calculated by windows of 300 pb for analyzed Tephritidae species, expected and normalized values.

Supplementary Table S7. Ka/Ks substitution values per protein coding gene by analyzed Tephritidae species.

Supplementary Table S8. Neutrality plot values per protein coding gene by analyzed Tephritidae species. Values for GC1, GC2, GC3 and average values from GC1 and GC2.

Supplementary Table S9. Dataset information of host/lifestyle and distribution of analyzed members of Tephritidae.
