# Supplementary material for: Mitochondrial Genome Variations and Possible Adaptive Implications in Some Tephritid Flies (Diptera, Tephritidae)
Source: Int J Mol Sci. 2025 Jun 10;26(12):5560. doi: 10.3390/ijms26125560 (PMC12193529; doi:10.3390/ijms26125560)
Supplement: Supplementary file 1 [file ijms-26-05560-s001.zip › Supplementary Table S1.pdf]

Sp	NCBI Accession	Length	Nucleotide composition(%)						
			**A**	**C**	**G**	**T**	**Ns**	**A+T**	**G+C**
Bactrocera tsuneonis	NC_038164	15865	38,3	22,4	11	28,3	0	66,6	33,4
Bactrocera minax	NC_014402	16043	38	21,5	11,2	29,2	0	67,2	32,7
Bactrocera rubigina	NC_046521	15285	39,3	16,9	10,1	33,8	0	73,1	27
Bactrocera melastomatos	NC_029467	15954	39,6	16,4	9,8	34,2	0	73,8	26,2
Bactrocera ruiliensis	NC_046952	15870	39,2	16,2	10,3	34,4	0	73,6	26,5
Bactrocera thailandica	NC_053983	15915	39,2	16,1	10,3	34,4	0	73,6	26,4
Bactrocera carambolae	NC_009772	15915	39,2	16,2	10,3	34,4	0	73,6	26,5
Bactrocera dorsalis	NC_008748	15915	39,3	16,2	10,2	34,3	0	73,6	26,4
Bactrocera neohumeralis	NC_062139	15924	38,8	16,9	10,6	33,7	0	72,5	27,5
Bactrocera tryoni	NC_014611	15925	38,8	16,9	10,7	33,7	0	72,5	27,6
Bactrocera correcta	NC_018787	15936	38,9	16,4	10,4	34,3	0	73,2	26,8
Bactrocera zonata	NC_027725	15935	39,1	16,3	10,4	34,3	0	73,4	26,7
Bactrocera frauenfeldi	NC_062138	15935	39,4	15,9	10,1	34,5	0	73,9	26
Bactrocera arecae	NC_028327	15900	39,1	17,5	10,3	33,2	0	72,3	27,8
Bactrocera limbifera	NC_037722	15860	39,3	16,8	10,1	33,7	0	73	26,9
Bactrocera ritsemai	NC_037723	15927	39,3	17,9	10,2	32,6	0	71,9	28,1
Bactrocera umbrosa	NC_029468	15898	38,2	18,4	11,2	32,3	0	70,5	29,6
Bactrocera latifrons	NC_029466	15977	38,7	18,3	10,6	32,4	0	71,1	28,9
Bactrocera biguttula	NC_042712	15829	39,3	17,2	9,9	33,6	0	72,9	27,1
Bactrocera oleae	NC_005333	15815	39,5	17,5	9,9	33,1	0	72,6	27,4
Dacus axanus	BK062949*	15772	39,2	17,5	9,7	32,4	1,2	71,6	27,2
Dacus conopsoides	NC_043843	15852	39,8	18,1	9,8	32,3	0	72,1	27,9
Dacus longicornis	NC_032690	16253	39,8	17,9	9,8	32,5	0	72,3	27,7
Dacus haikouenis	MZ087939	15798	37,6	16,6	9,5	32	0	69,6	26,1
Dacus trimacula	NC_053984	15847	39,1	17	10,2	33,7	0	72,8	27,2

Dacus vijaysegarani	NC_061932	15886	39,3	17	10,1	33,7	0	73	27,1
Dacus bivittatus	NC_046468	15833	39,4	16,9	9,9	33,9	0	73,3	26,8
Zeugodacus scutellaris	NC_061658	15931	39	16,5	10,4	34,1	0	73,1	26,9
Zeugodacus proprediaphora	NC_049063	15829	39,5	16,3	9,8	34,3	0	73,8	26,1
Zeugodacus diaphorus	NC_028347	15890	39,5	16,1	9,8	34,7	0	74,2	25,9
Zeugodacus scutellatus	NC_027254	15915	39	16,9	10	33,6	0,5	72,6	26,9
Zeugodacus strigifinis	NC_062140	15858	39,8	16,6	10,1	33,6	0	73,4	26,7
Zeugodacus cucurbitae	NC_016056	15825	38,1	16,4	10,7	34,7	0	72,8	27,1
Zeugodacus tau	NC_027290	15687	38,8	16,3	10,4	34,4	0	73,2	26,7
Zeugodacus cilifer	NC_052852	15843	39,7	16,1	9,9	34,3	0	74	26
Felderimyia fuscipennis	NC_052851	16536	38,2	17,5	10,3	34	0	72,2	27,8
Ceratitis capitata	NC_000857	15980	39,6	13,3	9,2	37,9	0	77,5	22,5
Ceratitis rosa	NC_053847	16047	39,5	13,5	9,1	37,9	0	77,4	22,6
Ceratitis fasciventris	NC_035497	16017	39,5	13,6	9,3	37,7	0	77,2	22,9
Ceratitis quilicii	NC_053846	16035	39,5	13,4	9,1	38	0	77,5	22,5
Anastrepha fraterculus	NC_034912	16739	41	13,8	9,1	36,1	0	77,1	22,9
Rhagoletis cerasi	NC_061399	16438	41,1	13,3	9,2	36,4	0	77,5	22,5
Rhagoletis batava	BK062952*	16351	38,5	12,6	8,7	35,1	5,2	73,6	21,3
Rhagoletis pomonella	BK062954*	16347	39,8	13,3	9,5	36,6	0,8	76,4	22,8
Rhagoletis zephyria	BK062955*	16362	37,6	12,7	9,2	34,7	5,8	72,3	21,9
Rhagoletis completa	BK062953*	16333	41,1	12,7	9	36,8	0,4	77,9	21,7
Carpomya incompleta	BK062947*	15818	37,7	13	9,4	34,6	5,5	72,3	22,4
Carpomya vesuviana	BK062948*	15814	37,4	13,3	9,5	34,2	5,5	71,6	22,8
Acidiella diversa	NC_053982	15531	40,1	12,6	9,4	37,9	0	78	22
Euleia heraclei	BK062950*	15899	40,6	12,1	9,2	37,7	0	78,3	21,3
Tephritis femoralis	NC_047184	15117	41,7	11,6	8,5	38,2	0	79,9	20,1
Tephritis californica	BK062956*	15056	41,6	11,6	8,5	38,1	0,2	79,7	20,1
Merzomyia westermanni	BK062951*	15911	41,9	13,3	8,2	36,5	0	78,4	21,5

Procecidochares utilis	NC_020463	15922	41,9	11,3	7,9	38,9	0	80,8	19,2
